# The E/e’ Ratio—Role in Risk Stratification of Acute Heart Failure with Preserved Ejection Fraction

**DOI:** 10.3390/medicina57040375

**Published:** 2021-04-13

**Authors:** Marilena-Brîndușa Zamfirescu, Liviu-Nicolae Ghilencea, Mihaela-Roxana Popescu, Gabriel Cristian Bejan, Sean Martin Maher, Andreea-Catarina Popescu, Maria Dorobanțu

**Affiliations:** 1 Cardiothoracic Pathology Department, Carol Davila University of Medicine and Pharmacy, 020021 Bucharest, Romania; brindusa.zamfirescu@gmail.com (M.-B.Z.); crrsty1@yahoo.com (G.C.B.); andreea.popescu@umfcd.ro (A.-C.P.); maria.dorobantu@gmail.com (M.D.); 2 Department of Cardiology, Elias Emergency University Hospital, 011461 Bucharest, Romania; 3 Department of Accidents and Emergencies, St. Vincent University Hospital, D04 N2E0 Dublin 4, Ireland; seanmahercaroldavila@gmail.com; 4 Department of Cardiology, Clinic Emergency Hospital, 20322 Bucharest, Romania

**Keywords:** acute heart failure, preserved ejection fraction, E/e’ ratio, predictor, prognosis, heart failure, rehospitalization, echocardiography

## Abstract

*Background and Objectives*: Heart failure with preserved ejection fraction (HFpEF) remains a worldwide management problem. Although there is a general effort for characterizing this population, few studies have assessed the predictive value of the echocardiographic E/e’ ratio in patients with acute HFpEF. The aim of the study was to identify groups with different prognosis in patients hospitalized with a first acute episode of HFpEF. *Materials and Methods*: The primary endpoint of the study was heart failure readmissions (HFR) at 6 months, while the secondary outcome was six-month mortality. We consecutively enrolled 91 patients hospitalized for the first time with acute HFpEF. We examined the E/e’ ratio as an independent predictor for HFR using univariate regression. *Results*: We identified and validated the E/e’ ratio as an independent predictor for HFR. An E/e’ ratio threshold value of 13.80 was calculated [(area under the receiver operating characteristic curve (AUROC) = 0.693, sensitivity = 78.60%, specificity = 55%, *p* < 0.004)] and validated as an inflection point for an increased number of HFR. Thus, we divided the study cohort into two groups: group 1 with an E/e’ ratio < 13.80 (n = 39) and group 2 with an E/e’ ratio > 13.80 (n = 49). Compared to group 1, group 2 had an increased number of HFR (*p* = 0.003) and a shorter time to first HFR (*p* = 0.002). However, this parameter did not influence all-cause mortality within six months (*p* = 0.84). *Conclusions*: The dimensionless E/e’ ratio is a useful discriminator between patients with acute HFpEF. An E/e’ value over 13.80 represents a simple, yet effective instrument for assessing the HFR risk. However, all-cause mortality at six months is not influenced by the E/e’ ratio.

## 1. Introduction

Heart failure with preserved ejection fraction (HFpEF) is a distinct heterogenous syndrome with multiple etiologies, characterized by increased cardiac filling pressures [1]. Non-invasive estimation of left ventricular diastolic function is an important goal of echocardiographic examination in HFpEF. However, it is unclear which parameter best predicts the outcomes and provides the most appropriate instrument for risk stratification [2,3,4]. This is because several gaps still exist in our knowledge of HFpEF: diagnostic gaps (optimal cut-points for ejection fraction and natriuretic peptides, better characterization of varying subsets of disease with different underlying pathophysiology), demographic gaps (pathophysiological basis for preponderance in women and elderly), treatment gaps, and mortality patterns.

Although the prognosis of HFpEF is nearly as poor as that of heart failure with reduced ejection fraction (HFrEF), the determinants of prognosis remain insufficiently defined [5,6]. HFpEF remains a condition in which no treatment has yet been shown convincingly to reduce morbidity and mortality [7,8,9,10,11]. This lack of therapeutic options relates to the pathophysiologic complexity of HFpEF, as it is not a uniform disease. In order to make therapeutic progress we have to improve patient phenotype characterization and to identify specific patient groups who could benefit from tailored treatment.

The need for rehospitalization of patients after an acute episode of HFpEF is a turning point in the evolution of the disease, indicating a worsening prognosis [12,13]. This stage is the optimal moment for assessing certain patient characteristics, which will contribute to a better understanding of HFpEF events and progression. Patients estimated to be at high-risk may require more observation and management, while those considered to be at low-risk may be managed with less exhaustive monitoring and therapies.

Is this syndrome only a collection of comorbidities? The prognosis of patients with HFpEF is substantially worse than that of patients with hypertension, diabetes, and other conditions that increase cardiovascular risk. The lessons of previous trials are that HFpEF is not just about old age, female gender, and high blood pressure [8,14,15,16].

The E/e’ ratio is a parameter for diastolic function assessment that is frequently used for HFpEF evaluation. It is feasible, and its values can guide clinical care [17]. However, the E/e’ ratio interval between 8 and 15 still represents a gray area for diastolic dysfunction [18]. More data regarding the prognostic value of E/e’ in acute HFpEF is needed.

In our study, we sought to outline the echocardiographic E/e’ ratio phenotype of the patients with an acute HFpEF episode, and to identify the value that increases the risk for medium term heart-failure rehospitalizations.

## 2. Materials and Methods

### 2.1. Study Design

This prospective observational study was initiated in April 2017 at Elias University Hospital (EUH). It is based on the analysis of a group of 91 patients hospitalized for an initial acute decompensation of HFpEF. Demographic, clinical, biochemical, and echocardiographic characteristics were recorded at baseline and used for further analysis.

### 2.2. Inclusion Criteria

The inclusion criteria were: acute dyspnea of cardiac origin as the main symptom at the emergency department, requiring hospitalization for the first time, left ventricular ejection fraction (LVEF) ≥ 50% [1] (assessed by echocardiography with the modified Simpson’s rule), N-terminal pro-B type natriuretic peptide (NT-proBNP) > 300 pg/mL (in sinus rhythm) and > 600 pg/mL (in atrial fibrillation) [19,20] and at least one additional criterion [left ventricle mass index (LVMI) ≥ 115 g/m² for males and ≥95 g/m² for females or diastolic dysfunction (defined as at least 3 of the following: average E/e’ > 14, septal e’ velocity < 7 cm/s or lateral e’ velocity < 10 cm/s, tricuspid regurgitation velocity > 2.8 m/s, left atrial volume index (LAVI) > 34 mL/m²)].

### 2.3. Exclusion Criteria

The exclusion criteria were: significant left heart valve disease (more than moderate mitral or aortic regurgitation, more than mild mitral or aortic stenosis), severe mitral annulus calcification, acute coronary syndromes, acute pulmonary embolism, pericardial constriction, chronic kidney disease stage IV or dialysis. Patients that were lost to follow-up were also excluded from the study.

The investigation conforms to the principles outlined in the Declaration of Helsinki [21] and was also approved by the ethics committee of EUH (protocol approval 9433/20.10.2016). All patients provided written informed consent.

### 2.4. Study Endpoints and Groups Definition

The primary endpoint was the number of heart failure rehospitalizations (HFR), while the secondary endpoint was mortality, both of them assessed at 6 months.

We aimed to define two groups with different prognosis based on the echocardiography diastolic function parameter E/e’ ratio.

### 2.5. In-Patient Assessment

On admission, we performed clinical and biological assessment for each patient, including medical history. Each patient further underwent standard two-dimensional echocardiography within the first 24 h of admission using a Vivid T8 Pro, GE Healthcare, according to recommendations of the American Society of Echocardiography and the European Association of Cardiovascular Imaging [18,22,23]. We assessed for all patients the ratio of early mitral inflow to tissue velocity of the mitral annulus (E/e’ ratio), the systolic (s) velocities, and early diastolic annular velocities of the septum and lateral wall using TDI. We used the average e’ velocity obtained by tissue Doppler imaging (TDI) from the septal and lateral mitral annulus [18]. For patients that had atrial fibrillation (AF) during the echocardiographic examination we used average velocity measurements from 10 consecutive cycles [18]. Echocardiographic measurements were performed offline by a cardiology specialist, blinded to the clinical data, then revised by an experimented investigator.

Prior history of coronary artery disease (CAD) was ascertained systematically using a combination of a self-reporting, electrocardiogram, review of all available prior medical records, and physician contact. Chronic kidney disease (CKD) was defined as an estimated glomerular filtration rate < 60 mL/min/1.73 m² [24] Diabetes mellitus (DM) was defined by treatment with antidiabetic medications or a haemoglobin A₁C ≥ 6.5 mg/dL. Obesity was defined by a body mass index (BMI) ≥ 30 kg/m².

Patients that were discharged were monitored either by scheduled visits and phone calls, or during readmissions to our hospital within six months.

### 2.6. Statistical Analysis

Data for continuous variables are presented as mean± standard deviation (SD) (%) when the distribution is uniform and as medians and interquartile range (IQR) for non-Gaussian distribution. Correlation between categorical variables used Spearman correlation (rS), and Pearson correlation (rP) was used for continuous variables.

A complete evaluation of E/e’ ratio performance was assessed for discrimination with the AUROC (area under the receiver operating characteristic curve) and for calibration with the Hosmer–Lemeshow goodness-of-fit test as well as the Nagelkerke pseudo-R2 test. We tested the E/e’ ratio as an independent predictor with an AUROC ≥ 0.650 and a Hosmer–Lemeshow *p* = value ≥ 0.05 [25]. Once it met these criteria, we calculated a cut-off according to the maximum Youden index. Cut-off characteristics were reported using sensitivity, specificity, positive predictive value (PPV), negative predictive value (NPV), accuracy, positive likelihood ratio (+LR), and negative likelihood ratio (−LR). Our results were validated by using a validation contingent of 44 patients which was randomized internally from the initial cohort. We analyzed the initial training cohort and the validation contingent by comparing the AUROC curves and by evaluating inter-ROC curve difference (with Hanley–McNeil test).

We performed comparisons of the central tendency of the baseline characteristics and endpoints of the groups using a Student *t*-test for normally distributed continuous variables, and nonparametric tests (Mann–Whitney U rank-sum test) to compare the non-normally distributed continuous variables. Categorical data are reported as numbers (percentages %), with group comparisons using Pearson’s chi-square test.

A Cox analysis was performed, and the results were expressed as hazard ratio to estimate the prognosis. All *p*-values were two-sided and we considered a *p*-value < 0.05 to be statistically significant. The statistical analysis was performed with the SPSS (Statistical Package for the Social Sciences) program, version 21 software (IBM SPSS Statistics, IBM Corp., Armonk, NY, USA).

## 3. Results

### 3.1. Patient Population

**Figure 1:**
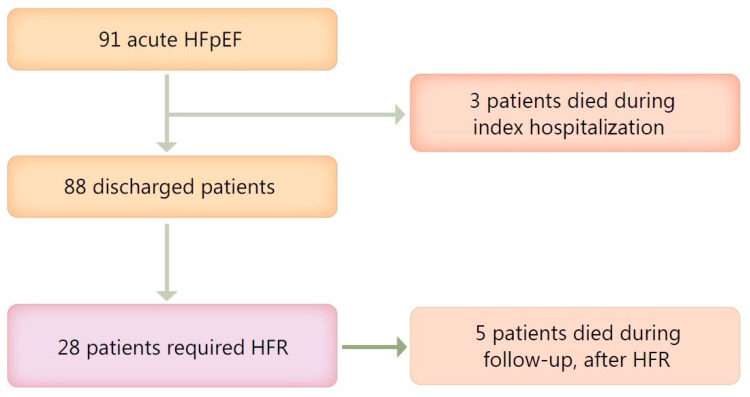
Patient population flow-chart. HFpEF- heart failure with preserved ejection fraction; HFR-heart failure readmissions

The study population consisted of 91 patients hospitalized in EUH between April 2017 and October 2019 for a first episode of acute HFpEF. Eight patients died within the first 6-months and twenty-eight required HFR. Three patients out of those hospitalized died during the initial hospitalization, while 88 patients were discharged and continued follow-up for six months (see Figure 1).

### 3.2. Baseline Characteristics

The mean age of the patients was 73.04 ± 10.61 years, with predominantly female patients (68.10%). We encountered a high incidence of obesity (59.30%), arterial hypertension (100%), DM (56%), AF (69.20%), and CKD (43.9%), while 23.10% patients had CAD (with more than 50% stenosis, in more than one vessel). AF was persistent or permanent in 48.35% of patients and paroxysmal in 20.87% of patients. In addition, 57.14% of patients had AF during the echocardiographic examination. The patients were also diagnosed with additional conditions, such as chronic obstructive pulmonary disease (COPD) (13%), asthma (8.7%), sleep apnoea syndrome (13.20%), and cerebrovascular disease (19.80%) (see Table 1).

HFpEF is defined as an association of comorbidities, consequently, we have observed the presence of 11 comorbidities in the studied population: obesity, hypertension, hyperlipidemia, DM, history of stroke, CAD, AF, CKD, COPD or asthma, obstructive sleep apnoea, and anemia (defined as Hb level < 13 g/dL in men, <12 g/dL in women). Fifty-seven percent (n = 70) of the patients had at least five comorbidities: five (n = 18 patients), six (n = 14 patients), seven (n = 18 patients), eight (n = 11 patients), nine (n = 8 patients), ten (n = 1 patient). The remaining 21 patients had less than five comorbidities as follows: two (n = 1 patients), three (n = 8 patients), four (n = 12 patients).

We also identified a gender difference in the distribution of the comorbidities, with a predominance of diabetic women vs. men (67.7% vs. 41%). Also, women were more frequently dyslipidemic (87% vs. 72%) and obese (61.2% vs. 58.6%).

### 3.3. E/e’ Ratio—Independent Predictor for HFR Rehospitalization

The HFR within 6 months had a moderate correlation (r = 0.37, *p* = 0.001) with the E/e’ ratio, the highest correlation of all the echocardiographic characteristics, and was further analyzed, while the mortality at 6 months had a weak correlation with E/e’ ratio (r = −0.014, *p* = 0.84). The E/e’ ratio mean value for the entire cohort on admission was 14.51 ± 4.61 (95% CI = 13.55–15.47). The prediction of the E/e’ ratio for HFR at 6 months (*p*-value 0.022) was not influenced by the presence of DM, age, sex, dyslipidemia or BMI in which *p*-values were greater than 0.05 according to Cox regression.

**Figure 2:**
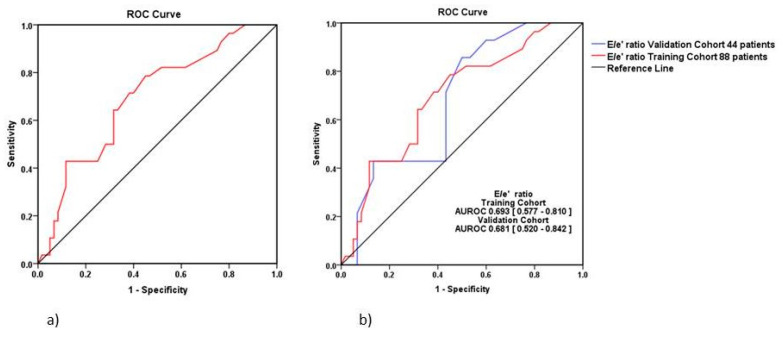
The ROC curve for HFR according to E/e’ ratio (prediction values from the regression equation) at 6 months from the first episode of HFpEF. (**a**) Training cohort (AUROC = 0.693, 95% CI = 0.577–0.810, *p* = 0.009); (**b**) Comparison between the training cohort (red) and the validation contingent (blue).

Also, according to the univariate regression, the E/e’ ratio was an independent predictor of HFR at 6 months after the first episode of acute HFpEF event, but not of mortality. The E/e’ ratio showed an AUROC ≥ 0.693 and a Hosmer–Lemeshow test *p* = 0.394, which meet the criteria for an independent predictor of HFR at 6 months (see Figure 2 and Table A1 and Table A2).

The regression equation for our model (E/e’ ratio) was calculated using the e-base natural logarithm of the odds ratio (OR) for HFR at 6 months:

Ln OR (for HFR at 6 months) = −2.92 + 0.144 × (E/e’ ratio)(1)

We performed an analysis of the E/e’ ratio in the training cohort (88 patients) based on its cut-off values in terms of either maximizing sensitivity and specificity, or according to the Youden’s Index criteria. The result was reported as follows: sensitivity = 78.50%, specificity = 55%, PPV = 44.90%, NVP = 84.60%, +LR = 1.75, −LR = 0.39 (see Table A3).

The validation of the results of the E/e’ ratio is displayed in Figure 2b. The two AUROC of the training cohort (0.693, 95% CI = 0.577–0.810) and the validation contingent (0.681, 95% CI = 0.520–0.842) showed close values without significant differences between them (Hanley & McNeil test, *p* = 0.879).

### 3.4. Echocardiographic E/e’ Ratio Cut-Off for HFR

**Figure 3:**
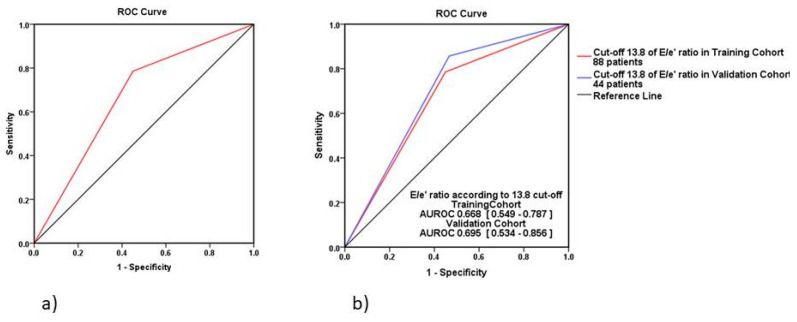
Receive operating curve of HFR at 6 months for E/e’ ratio. (**a**) ROC curve of HFR at 6 months for E/e’ ratio of 13.80 (n = 88); (**b**) ROC curves of HFR at 6 months for E/e’ ratio > 13.80 of the training cohort (n = 88, AUROC = 0.668) and validation contingent (n = 44, AUROC = 0.695). The two AUROC show close values and no significant differences among them (Hanley & McNeil test, *p* = 0.72).

The E/e’ ratio threshold value of 13.80 was identified with receiver operating characteristics (ROC) analysis for the early 6 months HFR after the first episode of acute HFpEF (AUC = 0.693, 95% CI = 0.577–0.810, sensitivity = 78.60%, specificity = 55%, *p* < 0.004) (see Figure 3). The study population was therefore divided into two groups: low E/e’ < 13.80 group (group 1, n = 39, 44.30%) and high E/e’ ratio > 13.80 group (group 2, n = 49, 55.70%).

According to the univariate analysis for the 13.80 cut-off of E/e’ ratio, patients with E/e’ ratio > 13.80 had a 4.48-fold odds ratio to be readmitted with heart failure within 6 months compared with patients with E/e’ ratio < 13.80 (95% CI = 1.590–12.63, *p* = 0.005), after a first episode of acute HFpEF.

We applied the pattern of the analysis of E/e’ ratio to the validation cohort internally randomized, with a 1:2.14 ratio between rehospitalized (n = 14) vs. non-rehospitalized (n = 30) patients as in the training cohort, and we noticed a comparable performance. The two AUROC of the training cohort and the validation contingent showed no significant differences (Hanley & McNeil test, *p* = 0.72) (Figure 3).

### 3.5. Clinical Outcomes

The details of all demographic and echocardiographic data, for both groups, are presented in Table 2. Several patient characteristics were found to differ significantly between the two groups.

Patients with diabetes had a 3.3-fold higher risk (OR) of having E/e’ ratio > 13.80 than non-diabetic patients (OR = 3.30, CI 95% = 1.37–7.94, *p* = 0.007). The biological data showed that hypercholesterolemic patients had a five-time higher risk of having an E/e’ ratio > 13.80 compared to patients without dyslipidemia (OR = 5, CI 95% = 1.46–17.07, *p* = 0.006). The oxygen saturation threshold value of 89.5% predicted with the highest sensitivity and specificity an E/e’ ratio value above 13.8 (sensitivity = 66.70%, specificity = 69.40, *p* = 0.04).

Interestingly, obese patients accounted for 73.3% of the readmissions, but only for 40% of the recorded deaths. This disparity between hospitalizations and mortality in obese patients with HFpEF was previously mentioned in other studies as the “obesity paradox”.

As already mentioned, our population was multi-morbid and we have found a moderate, statistically significant, correlation between the number of comorbidities and the number of HFR (r = 0.315; *p* = 0.002).

### 3.6. Echocardiography Parameters

The distribution of the study population in two groups yielded significant differences in some of the other echocardiographic parameters as well (see Table 2).

Parameters pertaining to the left ventricle, namely left ventricle mass (LVMI, g/m^2^), left ventricle end-diastolic diameter (LVEDD, mm) and left ventricle outflow tract time velocity integral (LVOT VTI, cm) were significantly increased in the high E/e’ group.

### 3.7. The Risk for Heart Failure Rehospitalization and All-Cause Mortality

The median duration (days) of the index hospitalization was not different among the two groups (8, IQR = 5 vs. 7, IQR = 6, *p* = 0.06), for E/e’ ratio > 13.80 and E/e’ ratio < 13.80, respectively. HFR was more frequent in group 2 (n = 22, 44.90%), compared with group 1 ((n = 6, 15.40%, *p* = 0.003). Also, there were more rehospitalizations per patient in group 2 (*p* = 0.003) (see Table 2).

All-cause mortality for the entire cohort during index hospitalization was 3.29%. An additional five patients (5.49%) died within the first six months. Thus, the all-cause mortality at six months reaches 8.78%. Mortality was similar across the two groups (*p* = 0.84) (see Table 2).

After six months of follow-up, the median time (days) until readmission was shorter in the E/e’ ratio > 13.80 group, (30, IQR = 7.50) compared to the E/e’ ratio < 13.80 group (75, IQR = 104.75) (*p* = 0.002).

**Figure 4:**
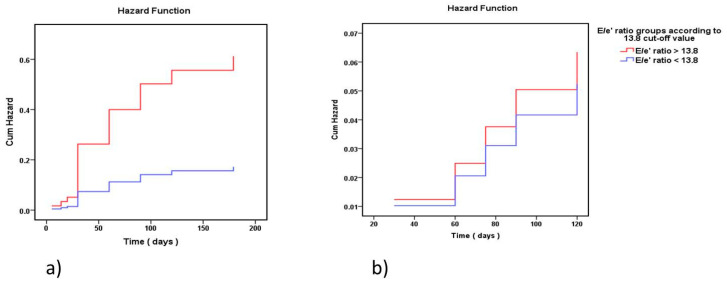
The risk curves for HFR (**a**) and mortality (**b**) in the 6-month follow-up period (Cum Hazard) according to Cox regression in the two groups: E/e’ ratio < 13.80 (blue), and E/e’ ratio > 13.80 (red) (time in days to readmission). The two curves are significantly apart for (**a**) HFR (*p* = 0.006) but not for (**b**) survival (*p* = 0.834).

The risk curves for the occurrence of HFR at 6 months (Cum Hazard) according to Cox regression show a statistically significant difference (*p*-value = 0.006), with more HFR in the group of patients with a high E/e’ ratio compared to the group with a low E/e’ ratio (see Figure 4a). Hazard ratio for HFR in the 6-month follow-up period after the first hospitalization in patients with an E/e’ ratio > 13.8 was 3.56 with 95% CI (1.441–8.796), *p =* 0.006. The risk curves for mortality in the 6-month follow-up period (Cum Hazard) according to Cox regression did not show any statistically significant difference (*p*-value = 0.834) between the two groups (see Figure 4b). Hazard ratio for mortality in the 6-month follow-up period after the first hospitalization in patients with HFpEF and E/e’ ratio > 13.8 compared to the group of patients with E/e’ ratio < 13.8 was 1.21 with 95% CI (0.202–7.244) (*p*-value 0.834).

A relative amount of 13.90% of HFR during the first 6 months of follow-up was predicted by our model, within the general population with acute HFpEF and an E/e’ ratio over 13.80 [Akaike information criterion (AIC) = 11.834, Bayesian information criterion (BIC) = 16.789].

## 4. Discussion

### 4.1. The Rationale for the Study

The purpose of our study, dedicated to the first episode of acute HFpEF, was focused on prognostication, while accepting that the singularity of the E/e’ as a marker might be a significant simplification. We aimed to reliably identify patients with acute HFpEF, at risk of hospital readmission after a 6 months follow-up, based on an easy to use, emergency department applicable echocardiographic parameter, and possibly detect a threshold value [26].

Most studies in the literature regarding HFpEF focus either on patients with chronic HFpEF or on acute heart failure as a whole, irrespective of EF. Our study, however, is dedicated to acute HFpEF and heart failure readmissions.

Since HFpEF is such a heterogeneous collection of disease phenotypes, the key to success is separating them further into more homogenous groups, based on certain characteristics that are accessible and reproducible.

### 4.2. Comparison to Similar Studies

The inclusion and exclusion criteria in our study have been very strict; therefore the resulting analyzed population is aligned with the current definitions of HFpEF [1,26,27]. In support of our selection criteria, a recent review concerning heart failure clinical trials concludes that only 27% of the HFpEF trials report patient-specific comorbidities, and unfortunately, most studies exclude a number of patients because of their comorbidities [28]. However, patients with DM, anemia, CKD, pulmonary disease were not excluded from our study, thus allowing a more realistic overview of this heterogeneous population. Along these lines, the multi-morbid population with accurately documented acute HFpEF was well represented and characterized. This is especially important since Khan et al. underline the need to also include a multi-morbid population in the HFpEF trials to create a broader image, closer to real-life cases. These combined pathologies bear the potential to induce acute presentations and heart failure decompensation [28]. In agreement with their publication we have observed in our study a moderate, although statistically significant, correlation between the number of comorbidities and HFR.

The proportion of women in our cohort was 68%, an observation which is in line with the results of other studies that mention a higher prevalence of female gender which seems to be one of the main distinguishing features of HFpEF from heart failure with reduced ejection fraction [29,30,31]. Dyslipidemia, obesity and DM in women, who are already more prone to inflammation than men, lead to accelerated endothelial dysfunction, ventricular fibrosis and hypertrophy, followed by chamber stiffness and diastolic dysfunction. DM doubles the risk to develop heart failure in diabetic women, as compared to men [29]. Indeed, our population was predominantly female and with a predominance of diabetic women vs. men (67.7% vs. 41%). Also, women were more frequently dyslipidemic (87% vs. 72%) and obese (61.2% vs. 58.6%). Dyslipidemia has a potential role in insulin resistance and myocardial stiffness [32]. Consequently, this comorbidity burden leads to more frequent rehospitalization (37% women vs. 20% men) and death (11.3% vs. 3.4%). Interestingly, more women had an E/e’ ratio > 13.8 (37% vs. 20%).

DM is a common hallmark in patients with HFpEF. Diabetes was present in 55% of the patients in our study, which is higher than the previously reported association (30–40%) [33]. Also, comparable to other studies, we found that the presence of diabetes in HFpEF patients correlates with hospital readmissions [34]. As previously mentioned, hyperglycemia contributes to cardiac stiffness and impaired cardiac relaxation and consequently to diastolic dysfunction [35].

Hypertension was present in 100% of our study population justifying the presence of concentric left ventricular hypertrophy (LVH) in 83.5% of the patients. This is where our data differs from the literature, which reports that approximately one-third to two-thirds of patients with HFpEF do not have LVH [36,37]. Also, in their studies, Katz and Shah detect that a low percentage (12% and 9% respectively) of the patients have an eccentric pattern of LVH rather than a concentric one [37,38].

A recent hypothesis, entitled the “obesity paradox”, states that although obese patients with cardiovascular disease have a higher risk of rehospitalization, their mortality risk is lower compared to normal-weight patients [39]. The same seems to be true for our patients who were obese (BMI > 30) in 63.71% of the cases and were characterized by a rehospitalization rate of 73.3%, yet a mortality of only 40%. Also, NT-proBNP is lower in obese patients than in patients with normal BMI. In the high E/e’ group there were 30 patients with obesity with a mean BMI = 32.63 and in the low E/e’ group there were 24 obese patients with a mean BMI = 31.51. This partly explains the fact that there is no significant difference in NT-proBNP between the two groups [40,41,42].

Our population characteristics (female predominance, obesity, hyperlipidemia, DM, anemia and renal insufficiency) overlap with high risk subgroups from a study on the I-PRESERVE study population [43]. What remains to be seen is if our long-term outcomes will match their findings.

Interestingly, the values for left atrial volume index (LAVI) and pulmonary artery systolic pressure (PAPs) did not differ significantly between groups. The values for LAVI are very high for group 1 and group 2 as well (mean value = 50.41 mL/m^2^ vs. 52.03 mL/m^2^). Although there is no significant difference between the two groups, LAVI is a little bit higher in the high E/e’ group. Also, LAVI is correlated with atrial fibrillation, that was equally distributed in the two groups. The fact that PAPS is not significantly different may have to do with the fact that all patients are at their first acute hospitalizations for HFpEF.

Previous studies searched for a cut-off that best predicts high left ventricular filling pressures [4,18,44]. Despite an inconstant correlation with increased filling pressures [45,46,47], some studies found that the E/e’ ratio has prognostic value in HFpEF [3,48,49]. Increased E/e’ was shown to be correlated with left ventricular stiffness, which is mediated by increased myocardial fibrosis—a known prognostic factor for major adverse cardiac events (MACE) [50]. The degree of myocardial fibrosis as evaluated through cardiac magnetic resonance (CMR) correlates well with diastolic dysfunction as assessed through E/e’ ratio [51]. Myocardial fibrosis and diastolic dysfunction evaluated through E/e’ is present even before LVH occurrence in hypertensive patients [52]. Regression of myocardial fibrosis, even in the absence of LVH regression, is accompanied by an improvement in LV diastolic function, which proves once more the correlation between fibrosis and E/e’ [53]. Thus increased E/e’ ratio may precede increased filling pressures. Although all of our study population was 100% hypertensive, it is a known fact that hypertensive cardiomyopathy evolves differently [54]. Thus, an increased E/e’ ratio identifies a group of patients with myocardial fibrosis, and therefore with unfavorable outcomes.

Most of the studies were performed on patients with chronic, not acute HFpEF. Our study focuses on the prognostic value of the E/e’ ratio, not on the estimation of filling pressures. Similarly, Blanco et al. found a prognostic cut-off of 14 for the E/e’ ratio. However, their study used the discharge assessment for the statistical analysis, not the admission evaluation [55]. The fact that they reported a very close cut-off value to the one we calculated (13.8) is indeed intriguing, as there seems to be little difference between the admission and discharge prognostic cut-off value of E/e’. Also, Blanco et al. found that a >50% reduction in admission E/e’ was correlated with better outcomes. Therefore, patients with worse prognosis have a constantly higher E/e’ ratio. Also, a sub-analysis of the KaRen study found that increased E/e’ values at 4–8 weeks after discharge have prognostic value [48]. The fact that our study found a prognostic cut-off right from admission underscores the purpose of the study, which is finding a tool that is readily available and easy to use, from the moment of patient’s presentation. Other studies looking into the prognostic value of E/e’ did not calculate a cut-off value, while others looked only for MACE and not specifically for HFR. We centered our study on HFR, as a paramount event in the evolution of the patient with HFpEF [13]. As a distinctive characteristic of our study population, the patients included in the study were hospitalized for the first time for an acute HFpEF decompensation. Thus, their characterization and evolution might offer a better perspective of the progression timeline of HFpEF from an early stage. Moreover, we have chosen the time of admission for echocardiographic evaluation, as Gheorghiade et al. indicated the admission parameters as possible predictors in heart failure [56,57]. We also looked into the specific differences between the two groups separated by the calculated E/e’ cut-off of 13.8, in order to get a clearer picture of what particular characteristics set them apart.

We demonstrated that for a cohort of in-patients with acute HFpEF, the E/e’ ratio is an independent predictor of HFR at 6 months. It is important to outline that the E/e’ ratio did not predict mortality at six months. Thus, the threshold of 13.80 is a good discriminator for HFR, but not for mortality at six months. Similar to our research, a recent study found that the predictors for rehospitalization and mortality in heart failure are not related [58].

It is our obvious conclusion that rehospitalization and all-cause mortality are not forecasted by the same predictors. In our opinion, this means rehospitalization and mortality have different pathological mechanisms, as acute events of decompensation and need for rehospitalization are based more on left ventricle burden, with the retrograde pulmonary barrier as a “trade-off” for the preservation of right ventricle function.

We consider that our results bring important insights into the applicability of the E/e’ ratio to patients with a first acute HFpEF event. This finding may have broad applicability in the assessment of patients with first acute HFpEF events, a value of 13.80 identifying the patients with higher risk of repeating acute events and needing rehospitalization within 6 months. The identified high-risk patients may benefit from closer monitoring and tailored management.

Perhaps E/e’ should be reappraised as a prognostic marker rather than a standalone diagnostic one. Our study backs the idea that it is not yet the time to declare the E/e’ ratio obsolete. We only have to be smarter about when and how we use it. The tools for diagnosis, prognosis and monitoring may, however, be different. It cannot be used for diagnostic purposes as a single indicator of diastolic function, but rather in conjunction with all the other parameters. This might be explained by the fact that increased E/e’ has more to do with myocardial stiffness than to the varying hemodynamic adaptation of the pulmonary capillary [59,60]. While using this single parameter for diagnosis might be an oversimplification, it might fit the purpose of prognostic marker [48].

In summary, the E/e’ ratio is an important parameter for the medium term prognosis after the first hospitalization for acute HFpEF. The value over 13.80 is a predictor for worse outcomes, with more rehospitalizations and a shorter time to first readmission, therefore the dimensionless E/e’ ratio is a good candidate for the role of discriminator between patients with acute HFpEF and providing prognostic information.

### 4.3. Study Limitations

The relatively small number of patients enrolled was the most important limitation. Several factors influence the discriminative power of E/e’ ratio: i.e., mitral E velocity is challenging to assess in patients with AF or moderate mitral annular calcifications, and E, as well as e’ normally decrease with age [3,44,61].

## 5. Conclusions

The dimensionless E/e’ ratio is a validated echocardiographic predictor for 6 months HFR after the first acute HFpEF event. The values over 13.80 predict a worse prognosis. All-cause mortality at six months is not influenced by the E/e’ ratio.

## Figures and Tables

**Table 1:** Key baseline characteristics of the patients with Acute Heart Failure with preserved ejection fraction (HFpEF) (n = 91).

Characteristics	Value
Number (%)	91 (100%)
Length of in-hospital stay, days, median (IQR)	7.50 (5)
Age at diagnosis, yo, mean ± SD (95% CI)	73.04 ± 10.61 (70.83–75.25)
Female gender, n (%)	62 (68.1%)
*Cardiovascular risk factors*	
High blood pressure, n (%)	91 (100%)
Diabetes mellitus, n (%)	51 (56%)
Tobacco smoking (current or former), n (%)	25 (27.5%)
Hypercholesterolemia, n (%)	75 (82.4%)
BMI, mean ± SD (95% CI)	32.13 ± 6.33 (30.81–33.45)
*Previous medical history*	
Medical history of CAD, n (%)	21 (23.1%)
Medical history of MI, n (%)	13 (14.3%)
Medical history of stroke, n (%)	18 (19.8%)
History of Atrial fibrillation, n (%)	63 (69.2%)
Medical history of lung disease, n (%)	44(48.40%)
Medical history of sleep apnoea, n (%)	12 (13.2%)
*Assessment on admission*	
Non-Invasive ventilation on admission, n (%)	20 (22%)
Mechanical ventilation on admission, n (%)	6 (6.6%)
Peripheral edema on admission, n (%)	53 (58.20%)
SaO_2_ on admission, median (IQR)	89 (6)
HR on admission, median (IQR)	96 (55)
SBP, mm Hg, mean ± SD (95% CI)	185.44 ± 34.77 (178.20–192.68)
Serum natremia, mmol/L, median (IQR)	140 (5)
eGFR, mL/min/1.73sqm, mean ± SD (95% CI)	66.52 ± 28.76 (60.53–72.51)
Hb, g/dL, mean ± SD (95% CI)	11.97 ± 2.03 (11.55–12.39)
NTproBNP, ng/L, median (IQR)	3074 (5241)

BMI: body mass index, CAD: Coronary Artery Disease, CI: confidence interval, eGFR: estimated glomerular filtration rate, Hb: Hemoglobin, HR: heart rate, IQR: interquartile range, MI: Myocardial Infarction, n: number, NTproBNP: N-terminal pro-B type natriuretic peptide SaO_2_: arterial oxygen saturation, SBP: systolic blood pressure, SD: standard deviation.

**Table 2:** Key baseline clinical, biological and echocardiographic characteristics at admission in the two groups. Clinical outcomes, HFR and all-cause mortality in the two groups.

Characteristics	E/e’ Ratio < 13.80	E/e’ Ratio > 13.80	*p*-Value
Number (%)	39 (44.30%)	49 (55.70%)	
Age (yo) mean ± SD (95% CI)	73.62 ± 9.95 (70.39–76.84)	72.35 ± 11.36 (69.08–75.61)	0.58 ^a^
Male gender, n (%)	12 (30.80%)	17 (34.7%)	0.69 **
Smoking status, n (%)	10 (25.60%)	15 (30.60%)	0.60 **
Medical history of DM, n (%)	15 (38.50%)	33(67.3%)	0.007 **
Medical history of hypercholesterolemia, n (%)	27 (69.20%)	45 (91.8%)	0.006 *
Medical history of CAD, n (%)	6 (15.40%)	15 (30.60%)	0.096 *
Medical history of MI, n (%)	3 (7.70%)	10 (20.40%)	0.095 *
Medical history of lung disease, n (%)	20 (51.30%)	22 (44.90%)	0.55 *
Medical history of sleep apnoea, n (%)	3 (7.70%)	8 (16.30%)	0.22 *
Pulmonary edema on admission, n (%)	9 (23.70%)	18 (37.50%)	0.17 *
Peripheral edema on admission, n (%)	23 (59%)	29 (59.20%)	0.98 *
BMI (kg/m²), mean ± SD (95% CI)	31.13 ± 4.70 (29.61–32.66)	32.73 ± 7.30 (30.63–34.83)	0.21 *
SpO_2_ (%) on index admission, median (IQR)	90 (5)	87 (6)	0.04 *
Heart rate (beats/min), mean ± SD (95% CI)	109.36 ± 35.35 (97.90–120.82)	104.73 ± 31.37 (95.72–113.75)	0.64 ^a^
SBP (mm Hg) on index admission, mean ± SD (95% CI)	176.41 ± 30.02 (166.68–186.14)	190.92 ± 37.27 (180.21–201.62)	0.046 ^a^
eGFR (mL/min/1.73 m²), mean ± SD (95% CI)	72.74 ± 25.27 (64.55–80.94)	61.38 ± 30.54 (52.61–70.16)	0.06 ^a^
Hb (g/dL) on index admission, mean ± SD (95% CI)	12.36 ± 1.59 (11.84–12.88)	11.70 ± 2.28 (11.04–12.35)	0.11 ^a^
NTproBNP (ng/L) on index admission, median (IQR)	3800 (6323)	2862 (5200)	0.41 *
Left ventricle ejection fraction (%), mean ± SD (95% CI)	55.87 ± 5.03 (54.24–57.50)	55.86 ± 6.70 (53.93–57.78)	0.99 ^a^
LVEDD (mm), mean ± SD (95% CI)	46.03 ± 5.15 (44.36–47.70)	49.02 ± 5.47 (47.45–50.59)	0.01 ^a^
Left ventricle mass (g/m^2^), median (IQR)	113 (26)	130 (45.50)	0.006 *
LVOT VTI (cm), median (IQR)	16 (8)	19.50 (5)	0.012 *
TAPSE < 17mm, n (%)	14 (35.90%)	8 (16.30%)	0.035 **
Systolic PAP (mm Hg), mean ± SD (95% CI)	40.46 ± 10.24 (37.14–43.78)	41.10 ± 18.16 (35.88–46.31)	0.83 ^a^
DBP (mm Hg) on index admission, mean ± SD (95% CI)	96.13 ± 15.41, (91.13–101.12)	101.22 ± 20.06, (95.46–106.99)	0.19 ^a^
Systolic PAP > 35, n (%)	30 (76.90%)	28 (57.10%)	0.052 **
IVC diameter > 21 mm, n (%)	20 (51.30%)	19 (38.80%)	0.24 **
IVC collapse < 50%, n (%)	16 (41%)	18 (36.70%)	0.68 **
LAVi (mL/m²), mean ± SD (95% CI)	50.41 ± 10.84 (46.90–53.92)	52.03 ± 13.08 (48.27–55.79)	0.53 ^a^
Length of in-hospital stay (days), median (IQR)	7 (6)	8 (5)	0.78 *
HFR at 6 months, n (%)	6 (15.40%)	22 (44.90%)	0.003 **
Time to first HFR (days), median (IQR)	75 (104.75)	30 (37.50)	0.002 *
Number of rehospitalization/patient at 6 months, median (IQR)	0 (1)	1 (2)	0.003 *
All-cause mortality at 6 months, n (%)	2 (5.1%)	3 (6.1%)	0.84 **

^a^*t*-test; * Mann–Whitney U test; ** Pearson Chi-Square test; BMI, body mass index; CAD, coronary artery disease; CI, confidence interval; DBP, diastolic blood pressure; DM, diabetes mellitus; eGFR, estimated glomerular filtration rate; Hb, hemoglobin; IQR, interquartile range; MI, myocardial infarction; NTproBNP, N-terminal pro-B-type natriuretic peptide; SBP, systolic blood pressure; SD, standard deviation; SpO₂, peripheral oxygen saturation; index, initial; LVEDD, left ventricular end-diastolic diameter; TAPSE, tricuspid annular plane systolic excursion; PAP, pulmonary artery pressure; RA, right atrium; IVC, inferior vena cava; LAVi, left atrial volume index; LVOT VTI, left ventricle outflow tract time velocity integral; HFR, heart failure readmission.

## Data Availability

All data is kept in hospital records, and is available on request.

## Appendix A

**Table A1:** The points of ROC curve model for values of E/e’ ratio according to rehospitalization for a first episode of acute HFpEF within the first 6 months after the first admission, for both the training cohort and the validation contingent.

Cut-Off Value Selection	Maximize Sensitivity Associated Criterion < 8.9		Maximize Specificity Associated Criterion > 27		Youden’s Inde xAssociated Criterion > 13.8	
	Training	Validation	Training	Validation	Training	Validation
Sensitivity (%)	100 (87.7–100)	100 (76.8–100)	0 (0–12.3)	0 (0–23.2)	78.57 (59–91.17)	85.71 (57.2–98.2)
Specificity (%)	13.33 (5.9–24.6)	10 (2.1–26.5)	100 (94–100)	100 (88.4–100)	55 (41.6.2–67.9)	50 (31.3–68.7)
PPV (%)	35 (32.8–37.3)	34.1 (31.5–36.9)	-	-	44.9 (36.7–53.4)	44.4 (34.5–54.8)
NPV (%)	100	100	68.2	68.2	84.6 (72.3–92.1)	88.2 (66.4–96.6)
+LR	1.15 (1–1.3)	1.11 (1–1.3)	-	-	1.75 (1.2–2.5)	1.71 (1.1–2.6)
−LR	0	0	1	1	0.39 (0.2–0.8)	0.29 (0.08–1.1)

PPV: positive predictive value; NPV: negative predictive value; +LR: positive likelihood ratio; −LR: negative likelihood ratio.

**Table A2:** Univariate Analysis for E/e’ ratio according to rehospitalization for a first episode of acute HFpEF within the first 6 months after the first admission.

Univariate Analysis	E/e’ Ratio
Coefficient	0.144
Coefficient-Standard Error	0.055
Coefficient-Significance	0.009
Intercept	−2.92
Intercept-Standard Error	0.872
Intercept-Significance	0.001
AUROC	0.693
Nagelkerke Pseudo-R²	0.117
Hosmer–Lemeshow *p*-value	0.394
AIC	70.892
BIC	75.847

AIC: Akaike information criterion; BIC: Bayesian information criterion; AUROC: area under the receiver operating characteristic curve.

**Table A3:** The points of ROC curve model for cut-off 13.80 of E/e’ ratio according to rehospitalization for a first episode of acute HfpEF within the first 6 months after the first admission, for both the training cohort and the validation contingent.

Cut-off Value Selection	Maximize Sensitivity Criterion ≥ 0		Maximize Specificity Criterion > 1		Youden’s Index Associated Associated Criterion > 0	
	Training	Validation	Training	Validation	Training	Validation
					Youden’s Index Associated-Value 0.335	Youden’s Index Associated-Value 0.39
Sensitivity	100 (87.7–100)%	100 (76.8–100)%	0 (0–12.3)%	0 (0–23.2)%	78.5 (59–91.7)%	85.71 (57.2–98.2)%
Specificity	0 (0–6)%	0 (0–11.6)%	100 (94–100)%	100 (88.4–100)%	55 (41.6- 67.9)%	53.33 (34.3–71.7)%
PPV	31.8%	31.8%	-	-	44.9 (36.7–53.4)%	46.2 (35.6–57.1)%
NPV	-	-	68.2%	68.2%	84.6 (72.3–92.1)%	88.9 (68–96.8)%
+LR	1	1	-	-	1.75 (1.32–2.5)	1.84 (1.2–2.8)
−LR	-	-	1	1	0.39 (0.2–0.8)	0.27 (0.07–1)

PPV: positive predictive value; NPV: negative predictive value; +LR: positive likelihood ratio; −LR: negative likelihood ratio.

## References

[1] Ponikowski P., Voors A.A., Anker S.D., Bueno H., Cleland J.G.F., Coats A.J.S., Falk V., González-Juanatey R., Harjola V.P., Jankowska E.A. (2016). 2016 ESC Guidelines for the diagnosis and treatment of acute and chronic heart failure. Eur. Heart J..

[2] Sharp A.S.P., Tapp R.J., Thom S.A.M.G., Francis D.P., Hughes A.D., Stanton A.V., Zambanini A., Brien E.O., Chaturvedi N., Lyons S. (2010). Tissue Doppler E/e′ ratio is a powerful predictor of primary cardiac events in a hypertensive population: An ASCOT substudy. Eur. Heart J..

[3] Mitter S.S., Shah S.J., Thomas J.D. (2017). A Test in Context: E/A and E/e′ to Assess Diastolic Dysfunction and LV Filling Pressure. J. Am. Coll. Cardiol..

[4] Lancellotti P., Galderisi M., Edvardsen T., Donal E., Goliasch G., Cardim N., Cardim N., Magne J., Laginha S., Hagendorff A. (2017). Echo-Doppler estimation of left ventricular filling pressure: Results of themulticentre EACVI Euro-Filling study. Eur. Heart J. Cardiovasc. Imaging.

[5] Owan T.E., Hodge D.O., Herges R.M., Jacobsen S.J., Roger V.L., Redfield M.M. (2006). Trends in prevalence and outcome of heart failure with preserved ejection fraction. N. Engl. J. Med..

[6] Lam C.S.P., Donal E., Kraigher-Krainer E., Vasan R.S. (2011). Epidemiology and clinical course of heart failure with preserved ejection fraction. Eur. J. Heart Fail..

[7] Massie B.M., Carson P.E., McMurray J.J., Komajda M., McKelvie R., Zile M.R., Anderson S., Donovan M., Iverson E., Staiger C. (2008). Irbesartan in patients with heart failure and preserved ejection fraction. N. Engl. J. Med..

[8] Cleland J.G.F., Tendera M., Adamus J., Freemantle N., Polonski L., Taylor J. (2006). The perindopril in elderly people with chronic heart failure (PEP-CHF) study. Eur. Heart J..

[9] Pitt B., Pfeffer M.A., Assmann S.F., Boineau R., Anand I.S., Claggett B., Clausell N., Desai A.S., Diaz R., Fleg J.L. (2014). Spironolactone for Heart Failure with Preserved Ejection Fraction. N. Engl. J. Med..

[10] Solomon S.D., McMurray J.J.V., Anand I.S., Ge J., Lam C.S.P., Maggioni A.P., Martinez F., Packer M., Pfeffer M.A., Pieske B. (2019). Angiotensin–Neprilysin Inhibition in Heart Failure with Preserved Ejection Fraction. N. Engl. J. Med..

[11] Garg R., Gorlin R., Smith T., Yusuf S. (1997). The Effect of Digoxin on Mortality and Morbidity in Patients with Heart Failure. N. Engl. J. Med..

[12] Pang P.S., Komajda M., Gheorghiade M. (2010). The current and future management of acute heart failure syndromes. Eur. Heart J..

[13] Gheorghiade M., Vaduganathan M., Fonarow G.C., Bonow R.O. (2013). Rehospitalization for heart failure: Problems and perspectives. J. Am. Coll. Cardiol..

[14] Seferovic P.M., Ponikowski P., Anker S.D., Bauersachs J., Chioncel O., Cleland J.G.F., Boer R.A., Drexel H., Gal T.B., Hill L. (2019). Clinical Practice Update on Heart Failure 2019: Pharmacotherapy, Procedures, Devices and Patient Management. An Expert Consensus Meeting Report of the Heart Failure Association of the European Society of Cardiology.

[15] McMurray J.J.V., Carson P.E., Komajda M., McKelvie R., Zile M.R., Ptaszynska A., Staiger C., Donovan J.M., Massie B.M. (2008). Heart failure with preserved ejection fraction: Clinical characteristics of 4133 patients enrolled in the I-PRESERVE trial. Eur. J. Heart Fail..

[16] Desai A.S., Lewis E.F., Li R., Solomon S.D., Assmann S.F., Boineau R., Clausell N., Diaz R., Fleg J.L., Gordeev I. (2011). Rationale and design of the Treatment of Preserved Cardiac Function Heart Failure with an Aldosterone Antagonist Trial: A randomized, controlled study of spironolactone in patients with symptomatic heart failure and preserved ejection fraction. Am. Heart J..

[17] Little W.C., Oh J.K. (2009). Echocardiographic evaluation of diastolic function can be used to guide clinical care. Circulation.

[18] Nagueh S.F., Smiseth O.A., Appleton C.P., Byrd B.F., Dokainish H., Edvardsen T., Flachskampf F.A., Gillebert T.C., Klein A.L., Lancellotti P. (2016). Recommendations for the Evaluation of Left Ventricular Diastolic Function by Echocardiography: An Update from the American Society of Echocardiography and the European Association of Cardiovascular Imaging. J. Am. Soc. Echocardiogr..

[19] Pieske B., Butler J., Filippatos G., Lam C., Maggioni APietro Ponikowski P., Shah S., Solomon S., Kraigher-Krainer E., Samano E.T., Scalise A.V. (2014). Rationale and design of the SOluble guanylate Cyclase stimulatoR in heArT failurE Studies (SOCRATES). Eur. J. Heart Fail..

[20] Lam C.S.P., Rienstra M., Tay W.T., Liu L.C.Y., Hummel Y.M., van der Meer P., de Boer R.A., Gelder I.C.V., Veldhuisen D.J.V., Voors A.A. (2017). Atrial Fibrillation in Heart Failure with Preserved Ejection Fraction: Association With Exercise Capacity, Left Ventricular Filling Pressures, Natriuretic Peptides, and Left Atrial Volume. JACC Heart Fail..

[21] (1997). World Medical Association Declaration of Helsinki: Recommendations Guiding Physicians in Biomedical Research Involving Human Subjects. JAMA J. Am. Med. Assoc..

[22] Lang R.M., Badano L.P., Victor M.A., Afilalo J., Armstrong A., Ernande L., Flachskampf F.A., Foster E., Goldstein S.A., Kuznetsova T. (2015). Recommendations for cardiac chamber quantification by echocardiography in adults: An update from the American Society of Echocardiography and the European Association of Cardiovascular Imaging. Eur. Heart J..

[23] Chetrit M., Cremer P.C., Klein A.L. (2020). Imaging of Diastolic Dysfunction in Community-Based Epidemiological Studies and Randomized Controlled Trials of HFpEF. JACC Cardiovasc. Imaging.

[24] Tromp J., Bamadhaj S., Cleland J.G.F., Angermann C.E., Dahlstrom U., Ouwerkerk W., Tay W.T., Dickstein K., Ertl G., Hassanein M. (2020). Post-discharge prognosis of patients admitted to hospital for heart failure by world region, and national level of income and income disparity (REPORT-HF): A cohort study. Lancet Glob. Health.

[25] Sambataro G., Giuffrè M., Sambataro D., Palermo A., Vignigni G., Cesareo R., Crimi N., Torrisi S.E., Vancheri C., Malatino L. (2020). The Model for Early COvid-19 Recognition (MECOR) Score: A Proof-of-Concept for a Simple and Low-Cost Tool to Recognize a Possible Viral Etiology in Community-Acquired Pneumonia Patients during COVID-19 Outbreak. Diagnostics.

[26] Pieske B., Tschöpe C., De Boer R.A., Fraser A.G., Anker S.D., Donal E., Edelmann F., Fu M., Guazzi M., Lam C.S.P. (2019). How to diagnose heart failure with preserved ejection fraction: The HFA-PEFF diagnostic algorithm: A consensus recommendation from the Heart Failure Association (HFA) of the European Society of Cardiology (ESC). Eur. Heart J..

[27] Van Aelst L.N.L., Arrigo M., Placido R., Akiyama E., Girerd N., Zannad F., Manivet P., Rossignol P., Badoz M., Sadoune M. (2018). Acutely decompensated heart failure with preserved and reduced ejection fraction present with comparable haemodynamic congestion. Eur. J. Heart Fail..

[28] Khan M.S., Samman Tahhan A., Vaduganathan M., Greene S.J., Alrohaibani A., Anker S.D., Vardeny O., Fonarow G.C., Butler J. (2020). Trends in prevalence of comorbidities in heart failure clinical trials. Eur. J. Heart Fail..

[29] Beale A.L., Meyer P.M.D., Marwick T.H., Lam C.S.P., Kaye D.M. (2018). Sex differences in cardiovascular pathophysiology why women are overrepresented in heart failure with preserved ejection fraction. Circulation.

[30] Gori M., Lam C.S.P., Gupta D.K., Santos A.B.S., Cheng S., Shah A.M., Claggett B., Zile M.R., Kraigher-Krainer E., Pieske B. (2014). Sex-specific cardiovascular structure and function in heart failure with preserved ejection fraction. Eur. J. Heart Fail..

[31] Ho J.E., Gona P., Pencina M.J., Tu J.V., Austin P.C., Vasan R.S., Kannel W.B., D’Agostino R.B., Lee D.S., Levy D. (2012). Discriminating clinical features of heart failure with preserved vs. reduced ejection fraction in the community. Eur. Heart J..

[32] Owei I., Umekwe N., Wan J., Dagogo-Jack S. (2016). Plasma lipid levels predict dysglycemia in a biracial cohort of nondiabetic subjects: Potential mechanisms. Exp. Biol. Med..

[33] Lindman B.R., Dávila-Román V.G., Mann D.L., McNulty S., Semigran M.J., Lewis G.D., Fuentes L.d.I., Joseph S.M., Vader J., Hernandez A.F. (2014). Cardiovascular phenotype in HFpEF patients with or without diabetes: A RELAX trial ancillary study. J. Am. Coll. Cardiol..

[34] Arora S., Lahewala S., Hassan Virk H.U., Setareh-Shenas S., Patel P., Kumar V., Tripathi B., Shah H., Patel V., Gidwani U. (2017). Etiologies, Trends, and Predictors of 30-Day Readmissions in Patients With Diastolic Heart Failure. Am. J. Cardiol..

[35] Habibi J., Aroor A.R., Sowers J.R., Jia G., Hayden M.R., Garro M., Barron B., Mayoux E., Rector R.S., Whaley-Connell A. (2017). Sodium glucose transporter 2 (SGLT2) inhibition with empagliflozin improves cardiac diastolic function in a female rodent model of diabetes. Cardiovasc. Diabetol..

[36] Zile M.R., Gottdiener J.S., Hetzel S.J., McMurray J.J., Komajda M., McKelvie R., Baicu C.F., Massie B.M., Carson P.E., Investigators I.P. (2011). Prevalence and significance of alterations in cardiac structure and function in patients with heart failure and a preserved ejection fraction. Circulation.

[37] Katz D.H., Beussink L., Sauer A.J., Freed B.H., Burke M.A., Shah S.J. (2013). Prevalence, clinical characteristics, and outcomes associated with eccentric versus concentric left ventricular hypertrophy in heart failure with preserved ejection fraction. Am. J. Cardiol..

[38] Shah A.M., Shah S.J., Anand I.S., Sweitzer N.K., O’Meara E., Heitner J.F., Sopko G., Li G., Assmann S.F., McKinlay S.M. (2014). Cardiac structure and function in heart failure with preserved ejection fraction: Baseline findings from the echocardiographic study of the treatment of preserved cardiac function heart failure with an aldosterone antagonist trial. Circ. Heart Fail..

[39] Carbone S., Lavie C.J. (2020). Disparate effects of obesity on survival and hospitalizations in heart failure with preserved ejection fraction. Int. J. Obes..

[40] Madamanchi C., Alhosaini H., Sumida A., Runge M.S. (2014). Obesity and natriuretic peptides, BNP and NT-proBNP: Mechanisms and diagnostic implications for heart failure. Int. J. Cardiol..

[41] Daniels L.B., Maisel A.S. (2007). Natriuretic Peptides. J. Am. Coll. Cardiol..

[42] Anjan V.Y., Loftus T.M., Burke M.A., Akhter N., Fonarow G.C., Gheorghiade M., Shah G. (2012). Prevalence, clinical phenotype, and outcomes associated with normal B-type natriuretic peptide levels in heart failure with preserved ejection fraction. Am. J. Cardiol..

[43] Kao D.P., Lewsey J.D., Anand I.S., Massie B.M., Zile M.R., Carson P.E., McKelvie R.S., Komajda M., McMurray J.J.V., Lindenfeld J. (2015). Characterization of subgroups of heart failure patients with preserved ejection fraction with possible implications for prognosis and treatment response. Eur. J. Heart Fail..

[44] Abudiab M.M., Chebrolu L.H., Schutt R.C., Nagueh S.F., Zoghbi W.A. (2017). Doppler Echocardiography for the Estimation of LV Filling Pressure in Patients with Mitral Annular Calcification. JACC Cardiovasc. Imaging.

[45] Donal E., Galli E., Fraser A.G. (2017). Non-invasive estimation of left heart filling pressures: Another nail in the coffin for E/e’?. Eur. J. Heart Fail..

[46] Sharifov O.F., Schiros C.G., Aban I., Denney T.S., Gupta H. (2016). Diagnostic accuracy of tissue Doppler index E/e’ for evaluating left ventricular filling pressure and diastolic dysfunction/heart failure with preserved ejection fraction: A systematic review and meta-analysis. J. Am. Heart Assoc..

[47] Hummel Y.M., Liu L.C.Y., Lam C.S.P., Fonseca-Munoz D.F., Damman K., Rienstra M., van der Meer P., Rosenkranz S., van Veldhuisen D.J., Voors A.A. (2017). Echocardiographic estimation of left ventricular and pulmonary pressures in patients with heart failure and preserved ejection fraction: A study utilizing simultaneous echocardiography and invasive measurements. Eur. J. Heart Fail..

[48] Donal E., Lund L.H., Oger E., Hage C., Persson H., Reynaud A., Ennezat P.V., Bauer F., Drouet E., Linde C. (2015). New echocardiographic predictors of clinical outcome in patients presenting with heart failure and a preserved left ventricular ejection fraction: A subanalysis of the Ka (Karolinska) Ren (Rennes) Study. Eur. J. Heart Fail..

[49] Holland D.J., Prasad S.B., Marwick T.H. (2010). Prognostic implications of left ventricular filling pressure with exercise. Circ. Cardiovasc. Imaging.

[50] Nakashima M., Sakuragi S., Miyoshi T., Takayama S., Kawaguchi T., Kodera N., Akai H., Koide Y., Otsuka H., Wada T. (2021). Fibrosis-4 index reflects right ventricular function and prognosis in heart failure with preserved ejection fraction. ESC Heart Fail..

[51] Moreo A., Ambrosio G., De Chiara B., Pu M., Tran T., Mauri F., Raman S.V. (2009). Influence of myocardial fibrosis on left ventricular diastolic function noninvasive assessment by cardiac magnetic resonance and echo. Circ. Cardiovasc. Imaging.

[52] Müller-Brunotte R., Kahan T., López B., Edner M., González A., Díez J., Malmqvist K. (2007). Myocardial fibrosis and diastolic dysfunction in patients with hypertension: Results from the Swedish Irbesartan Left Ventricular Hypertrophy Investigation versus Atenolol (SILVHIA). J. Hypertens..

[53] Brilla C.G., Funck R.C., Rupp H. (2000). Lisinopril-mediated regression of myocardial fibrosis in patients with hypertensive heart disease. Circulation.

[54] Drazner M.H. (2011). The progression of hypertensive heart disease. Circulation.

[55] Blanco R., Ambrosio G., Belziti C., Lucas L., Arias A., D’Antonio A., Oberti P., Carluccio E., Pizarro R. (2020). Prognostic value of NT-proBNP, and echocardiographic indices of diastolic function, in hospitalized patients with acute heart failure and preserved left ventricular ejection fraction. Int. J. Cardiol..

[56] Gheorghiade M., Zannad F., Sopko G., Klein L., Piña I.L., Konstam M.A., Massie B.M., Roland E., Targum S., Collins S.P. (2005). Acute heart failure syndromes: Current state and framework for future research. Circulation.

[57] Gheorghiade M., De Luca L., Fonarow G.C., Filippatos G., Metra M., Francis G.S. (2005). Pathophysiologic targets in the early phase of acute heart failure syndromes. Am. J. Cardiol..

[58] Voors A.A., Ouwerkerk W., Zannad F., van Veldhuisen D.J., Samani N.J., Ponikowski P., Ng L.L., Metra M., Maaten J.M.T., Lang C.C. (2017). Development and validation of multivariable models to predict mortality and hospitalization in patients with heart failure. Eur. J. Heart Fail..

[59] Kasner M., Westermann D., Steendijk P., Gaub R., Wilkenshoff U., Weitmann K., Hoffmann W., Poller W., Schultheiss H.P., Pauschinger M. (2007). Utility of Doppler echocardiography and tissue Doppler imaging in the estimation of diastolic function in heart failure with normal ejection fraction: A comparative Doppler-conductance catheterization study. Circulation.

[60] Kasner M., Gaub R., Sinning D., Westermann D., Steendijk P., Hoffmann W., Schultheiss H.P., Tschöpe C. (2010). Global strain rate imaging for the estimation of diastolic function in HFNEF compared with pressure-volume loop analysis. Eur. J. Echocardiogr..

[61] Dickinson M.G., Lam C.S., Rienstra M., Vonck T.E., Hummel Y.M., Voors A.A., Hoendermis E.S. (2017). Atrial fibrillation modifies the association between pulmonary artery wedge pressure and left ventricular end-diastolic pressure. Eur. J. Heart Fail..

